# Health Literacy and Sociodemographic Determinants of Cyberchondria: A Cross-Sectional Study Among Outpatients of a University Hospital in Turkey

**DOI:** 10.3390/healthcare13192445

**Published:** 2025-09-26

**Authors:** Cansu Özbaş, Enes Talha Yıldız, Hakan Tüzün, Ayşen Gülçin Kara Çiğdem, Asiye Uğraş Dikmen

**Affiliations:** Department of Public Health, Faculty of Medicine, Gazi University, Mevlana Boulevard, Yenimahalle, Ankara 06500, Türkiye

**Keywords:** cyberchondria, health literacy, online health information seeking, health promotion

## Abstract

**Background/Objectives**: This study aims to examine the relationship between health literacy, sociodemographic characteristics, and cyberchondria among individuals presenting to a university hospital. **Methods**: This cross-sectional study was conducted at the outpatient clinics of Gazi University Faculty of Medicine Hospital between 12 June and 16 June 2023. Individuals who reported using the internet for health-related information were included. The “Cyberchondria Severity Scale (CSS)” and the “Health Literacy Scale–Short Form” were used for data collection. Data were analyzed using descriptive statistics, correlation analysis, and multivariate regression tests with SPSS version 26.0. **Results**: A total of 965 participants with a mean age of 34.8 ± 11.8 years were included in the study, 55% of whom were female. Female gender (B = 4.095, CI: 1.67 to 6.52, *p* = 0.001) was significantly associated with higher levels of cyberchondria. In contrast, higher levels of health literacy (B = −0.329, CI: −0.47 to −0.19, *p* < 0.001) and better perceived health status (B = −3.065, CI: −4.65 to −1.48, *p* < 0.001) were significantly associated with lower levels of cyberchondria. **Conclusions**: The findings of this study demonstrate a significant negative association between health literacy and cyberchondria and highlight the importance of targeted health literacy interventions, particularly for women and individuals reporting poor perceived health. Furthermore, enhancing individuals’ skills in critically evaluating online health information and supporting digital literacy may help address cyberchondria.

## 1. Introduction

Health information-seeking behavior is a conscious process through which individuals seek knowledge about diseases, health risks, and health promotion [1]. Although healthcare professionals are considered the most reliable source in this process, the use of the internet as a source of health information has been steadily increasing [2,3]. Global studies indicate that a significant proportion of individuals use online resources to obtain health-related information. In an international study involving 12,000 participants across 12 countries, 75% of respondents reported using the internet to access health information [4]. While the internet promotes health information-seeking behavior through its easy, inexpensive, and fast access, it also poses risks regarding the accuracy and reliability of the information [5,6]. Various studies have shown that some online health information may be outdated, incomplete, or misleading. This can result in misinformation, delays in diagnosis and treatment processes, and even unnecessary utilization of healthcare services [7,8]. Particularly among individuals who frequently search for health-related information online, levels of health anxiety may increase. The literature reports that one in every five individuals who search for health information experiences an increase in health-related anxiety [9].

Continuous and repetitive health information seeking may lead to heightened anxiety, creating a vicious cycle. This condition is referred to in the literature as “cyberchondria.” Cyberchondria is defined as a process in which online health information searches amplify health anxiety, leading to more searches and consequently even greater anxiety. According to various studies, while a majority of the general population experiences moderate levels of cyberchondria, certain occupational groups report higher prevalence rates [10,11,12,13]. For instance, a study conducted among informatics sector workers in India found a cyberchondria prevalence of 55.6% [14]. Cyberchondria is not only a concern for individual health but also presents challenges for the efficiency and economic sustainability of healthcare services. Inaccurate or incomplete online information may lead individuals to undergo unnecessary medical tests, increase the frequency of physician visits, and contribute to an excessive workload on the healthcare system. Enhancing the reliability of online information sources, promoting conscious internet use, and supporting individuals’ critical appraisal skills may help mitigate cyberchondria [15,16,17].

Improving the reliability of online health information sources alone is insufficient in addressing cyberchondria. A meta-analysis in the literature has shown that health literacy interventions in primary healthcare services are associated with reductions in generalized anxiety and depression symptoms [18]. Health literacy—a multidimensional concept encompassing the ability to access, understand, evaluate, and use health-related information [19]—is crucial for how individuals engage with online health content. While adequate health literacy can help individuals make informed health decisions, limited literacy may leave them vulnerable to cyberchondria due to difficulty in evaluating the quality of the information encountered [20]. Research conducted in several European countries has shown that over 50% of adults have inadequate health literacy, which impairs their ability to manage their health appropriately [21].

In Turkey as well, limited health literacy poses a significant public health issue, resulting in adverse outcomes across a range of areas, from access to healthcare services to disease prevention. Studies conducted in the general population in Turkey have reported limited health literacy rates of 22.4% [22], 30.1% [23], and 40.1% [24], respectively. According to the Ministry of Health’s national study on health literacy levels and associated factors, 21.0% of the population had inadequate and 32.9% had problematic-limited health literacy [25]. Several studies conducted in different populations have demonstrated a negative association between health literacy and cyberchondria [11,26,27]. These findings suggest that initiatives aimed at improving health literacy may be associated with lower levels of cyberchondria [28]. As individuals improve their health literacy, they become better equipped to evaluate the credibility of online health information. They are therefore less likely to develop excessive health anxiety or unnecessary worry as a result of such searches [20].

Despite the growing literature on cyberchondria, most existing studies have focused either on general prevalence estimates or on its association with health anxiety and internet use habits. Only a limited number of investigations have directly examined how health literacy—an essential determinant of health-related decision-making—relates to cyberchondria. Furthermore, studies that have addressed this relationship often relied on bivariate analyses without adequately accounting for sociodemographic or health-related factors that may confound the association. To our knowledge, there is a lack of comprehensive evidence from Türkiye examining the interplay between health literacy, sociodemographic determinants, and cyberchondria using multivariate statistical approaches. Addressing this gap is important for examining the association between health literacy and cyberchondria in various population subgroups. Therefore, this study aims to comprehensively examine the relationship between cyberchondria and health literacy, along with sociodemographic factors.

## 2. Materials and Methods

### 2.1. Sampling

The study population consisted of individuals aged 18–65 who presented for any reason to the outpatient clinics of Gazi University Hospital—a tertiary care facility located in Ankara, the capital of Türkiye—between 12 June and 16 June 2023. This age range was chosen to minimize the influence of age-related differences in digital literacy, health perception, and internet use habits on the analysis. Xu and Starcevic (2025) reported that while cyberchondria levels tend to be higher in individuals aged ≥ 60, lower scores are observed among subgroups with inadequate digital literacy [29]. Similarly, previous studies investigating cyberchondria have generally included individuals within this age range [30,31]. Therefore, participant age was limited to 18–65 years in this study.

According to data obtained from the Statistics Unit of Gazi University Faculty of Medicine Hospital, the average number of weekly outpatient visits is approximately 7000, and this figure was accepted as the study population size. The sample size was calculated using OpenEpi v.3.01, with a 95% confidence interval, 50% assumed prevalence, design effect of 1.0, and a 3% margin of error. The required sample size was calculated as 927 participants, and with an added replacement rate of 25%, the final target was 1159 participants.

The inclusion criteria for the study were as follows: being between the ages of 18 and 65, and using the internet to seek health-related information. Individuals whose responses were inconsistent or incomplete and could not be included in the dataset during analysis were excluded from the study. Participants were selected using a convenience sampling method among those presenting to the outpatient clinics.

### 2.2. Implementation

This is an epidemiological study with a cross-sectional design. Data were collected through face-to-face interviews conducted between 12 June and 16 June 2023. Written administrative permission for the study was obtained from the Chief Physician’s Office of Gazi University Faculty of Medicine Hospital.

### 2.3. Data

In this study, data were collected using the questionnaire titled “Assessment of Sociodemographic Characteristics, Health-Related Features, Health Literacy, and Cyberchondria Levels among Individuals Presenting to a University Hospital Outpatient Clinic.” The questionnaire was developed by the research team conducting the study and consisted of a total of 55 items. The first five questions addressed sociodemographic characteristics (age, gender, marital status, educational level, and monthly household income). The following five questions focused on the participants’ and their families’ health-related conditions (tobacco and alcohol use, presence of chronic diseases in the participant or their family, and self-perceived health status). The subsequent 33 items were derived from the Cyberchondria Severity Scale (CSS), and the final 12 items were taken from the Short Form Health Literacy Scale (HLS-SF).

#### 2.3.1. Cyberchondria Severity Scale (CSS)

In the study, the “Cyberchondria Severity Scale (CSS)”, which will be used as the dependent variable, was developed by McElroy and Shevlin in 2014 to assess cyberchondria, a form of anxiety characterized by excessive online health research, and was later adapted into Turkish [32]. In the Turkish adaptation study, the Cronbach’s alpha coefficients calculated for the reliability of the subscales of the CSS ranged between 0.65 and 0.85; the Cronbach’s alpha coefficient for the entire scale was 0.89. The CSS consists of 33 items, is in a 5-point Likert format, and includes five sub-factors. These factors are: compulsion (8 items)—behavioral engagement in daily life as a result of online searches; distress (8 items)—excessive anxiety; excessiveness (8 items)—excessive and repetitive internet research on medical information; reassurance (6 items)—seeking relief while performing excessive searches for increasing adverse effects; and mistrust of medical professional (3 items)—distrust of medical professionals based on information obtained during repeated online searches. The scale is continuous, and the total cyberchondria severity score is calculated by summing the scores obtained from each item. The possible total score ranges from 33 to 165, and the higher the score, the higher the level of cyberchondria [10,32].

#### 2.3.2. Short-Form Health Literacy Scale (HLS-SF)

In the evaluation of the Short-Form Health Literacy Instrument, the formula (Index = (Mean − 1) × 50/3) is used. The mean is calculated by dividing the total scale score by the number of items on the scale. The index value calculated by this formula ranges from 0 to 50, with a higher score indicating better health literacy (2). The scale consists of 12 items with four-point Likert-type response options ranging from 1 (very difficult) to 4 (very easy) [33]. The Turkish validity and reliability of the scale were assessed on adults, and reliability was assessed using the Cronbach’s Alpha coefficient. The 12-item scale achieved a high reliability with a Cronbach’s Alpha coefficient of 0.856 [34].

Internal consistency was evaluated for the CSS and HLS-SF12 using Cronbach’s alpha. In the current sample, CSS showed excellent reliability (α = 0.93) for the total scale, with subscale alphas ranging from 0.67 to 0.95. HLS-SF12 demonstrated very good reliability (α = 0.878).

### 2.4. Statistical Analysis

In the descriptive statistics section, categorical variables were presented as counts and percentages, while continuous variables were reported as mean ± standard deviation. The normality of continuous variables was assessed using visual methods (histograms and probability plots) and analytical tests (Kolmogorov–Smirnov/Shapiro–Wilk tests). Data conforming to a normal distribution were expressed as mean ± standard deviation. Comparisons between two independent groups with normally distributed data were performed using Student’s *t*-test, while comparisons among more than two independent groups were conducted using one-way analysis of variance (ANOVA). Tukey’s HSD test was applied as a post hoc test to determine which groups differed significantly. Pearson’s correlation test was used to evaluate relationships between continuous variables after confirming normality. Correlation strength was interpreted based on Cohen’s (2013) guidelines, where r = 0.10, 0.30, and 0.50 indicate small, medium, and large correlations, respectively [35].

In this study, bivariate and multivariate models were constructed to identify factors associated with cyberchondria levels. The dependent variable in all linear regression models was the CSS score. Variables found to be statistically significant in the bivariate analysis were included in the multivariate model. In Model 1, health literacy score (HLS) was evaluated; in Model 2, health literacy score, age, gender, marital status, and educational level were included. In Model 3, variables found significant in Model 2, along with the presence of chronic disease and perceived health status, were assessed. The “stepwise” method was used as the variable selection approach in building regression models. A Type I error level of 0.05 was set. Statistical analyses were performed using IBM SPSS Statistics for Windows, version 26.0.

### 2.5. Ethical Approval

Gazi University Ethics Committee approved the study on 9 May 2023, with the research code 2023-680. Since the survey was conducted through face-to-face interviews, participants were verbally informed about the study, and their verbal consent was obtained prior to the interviews.

## 3. Results

**Figure 1:**
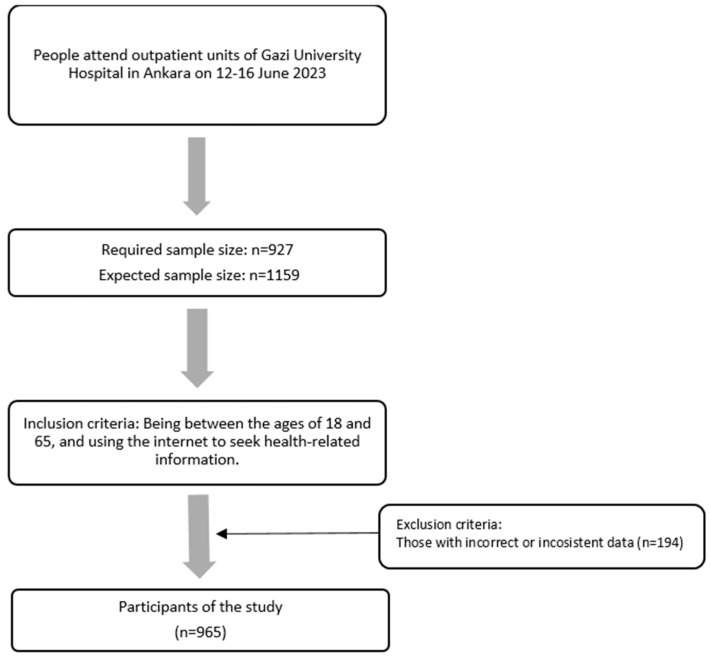
Study flowchart.

After excluding participants with inconsistent responses—i.e., those who left more than 10% of items blank or provided contradictory answers on similar items (*n* = 194)—the final analytic sample consisted of 965 participants. This number meets the originally calculated sample size before applying the replacement rate; therefore, the sample was considered statistically sufficient (Figure 1).

The participants’ mean age was 34.8 ± 11.8 years. When participants were asked about their reasons for consulting online sources regarding health, 839 (86.9%) stated it was to obtain information about diseases, 811 (84.0%) to understand the possible causes of symptoms (complaints), 616 (63.8%) to learn about treatment options, 382 (39.5%) to gather information about existing healthcare systems, and 323 (33.4%) to learn about others’ experiences with illness.

The mean CSS (Cyberchondria Severity Scale) score was 84.1 ± 19.6. The mean subscale scores were as follows: compulsion 14.2 ± 6.5, distress 20.6 ± 6.4, excessiveness 24.8 ± 5.9, reassurance 18.4 ± 4.5, and mistrust of medical professionals 6.1 ± 2.5. The mean score for the Health Literacy Scale was 32.5 ± 8.6.

In Table 1, the CSS (Cyberchondria Severity Scale) scores were compared according to certain descriptive characteristics of the participants. The CSS scores of women were found to be higher than those of men (*p* = 0.001). Participants who graduated from high school had higher CSS scores compared to university graduates (*p* = 0.002). Individuals with chronic illnesses had higher CSS scores than those without chronic conditions (*p* = 0.001). Participants who rated their health status as very poor/poor had higher CSS scores compared to those who rated their health as moderate, good, or very good (*p* < 0.001). Participants who reported using the internet most frequently to access health-related information had significantly higher CSS scores (*p* < 0.001).

### HL: Health Literacy; CSS: Cyberchondria Severity Scale

Table 2 presents the correlation between participants’ age and Health Literacy Scale (HLS) scores and Cyberchondria Severity Scale (CSS) scores. No significant correlation was found between age and CSS scores (*p* = 0.312), whereas a negative correlation was detected between HLS scores and overall CSS scores (*p* < 0.001). Similarly, negative correlations were observed between HLS scores and the CSS subdimensions of compulsion, distress, and mistrust of medical professionals (*p* < 0.001).

In Table 3 the factors associated with the level of cyberchondria were evaluated using a three-stage multivariate linear regression model. In the first model, only the health literacy variable was included, and it was found that each one-point increase in the Health Literacy Score (HLS) was significantly and negatively associated with the level of cyberchondria (B = −0.376; CI: −0.51 to −0.23; *p* < 0.001). The first model explained 2.7% of the variance in CSS (R^2^ = 0.027, adj. R^2^ = 0.026). In the second model, demographic variables (age, gender, marital status, and educational status) were added, and the gender variable was found to be significant—female participants had higher cyberchondria scores compared to male participants (B = 4.167; 95% CI: 1.72 to 6.61; *p* = 0.001). In contrast, age (*p* = 0.927), marital status (*p* = 0.727) and educational status (*p* = 0.273) were not significantly associated with cyberchondria. The second model explained 3.8% of the variance in CSS (R^2^ = 0.038, adj. R^2^ = 0.036). In the third and full model, the variables found significant in the second model were evaluated together with the presence of chronic illness and perceived health status. The negative association between health literacy and cyberchondria remained significant (B = −0.329; CI: −0.47 to −0.19 *p* < 0.001), and gender was also significantly associated with cyberchondria (B = 4.095; CI: 1.67 to 6.52; *p* = 0.001). Furthermore, a more positive perception of one’s general health status was significantly associated with lower levels of cyberchondria (B = −3.065; 95% CI: −4.65 to −1.48; *p* < 0.001), whereas the presence of chronic illness was not significantly associated with cyberchondria (*p* = 0.230). The third model explained 3.2% of the variance in CSS (R^2^ = 0.053, adj. R^2^ = 0.050).

Multicollinearity diagnostics indicated no concern, with Tolerance values ranging from 0.968 to 1.000 (all > 0.2) and VIF values ranging from 1.000 to 1.033 (all < 5).

## 4. Discussion

Cyberchondria has emerged in recent years as a concept in the literature, driven by the widespread use of digital technologies and the increasing reliance on the internet for seeking health-related information. Research in this field has also grown significantly in recent times. Identifying the factors that contribute to higher levels of cyberchondria in the population is of great importance for developing strategies to prevent and manage this condition.

### 4.1. The Association of Socio-Demographic Variables with Cyberchondria

In this study, female participants had significantly higher Cyberchondria Severity Scale (CSS) scores compared to males in both bivariate and multivariate analyses (B = 4.095, *p* = 0.001). Similar findings in the literature support these results [36,37,38,39]. This situation may be attributed to women’s greater concern about health-related issues, their stronger motivation to search for health information, and their tendency to express emotions more freely due to social role expectations. In this context, women’s more frequent and in-depth online information-seeking behaviors and may be associated with increased levels of cyberchondria. Developing targeted strategies to enhance women’s health literacy, considering these individual and societal tendencies, would be a constructive approach. In particular, actively involving women in health literacy programs, fostering skills for the critical evaluation of online health information, and supporting digital literacy competencies may be associated with lower levels of cyberchondria.

In this study, individuals with a higher education level (university degree or above) were found to have lower Cyberchondria Severity Scale (CSS) scores compared to those with only a high school education. In line with this finding, some studies have reported that increasing educational attainment is associated with decreased CSS scores, suggesting that individuals with lower education levels may have difficulty evaluating the quality of health information, which could lead them to search for health information online [11,31] repeatedly. Conversely, studies that found higher CSS scores among individuals with lower educational levels have attributed this to less frequent internet use or greater difficulties experienced while using the internet among those with lower education levels [40,41,42,43]. A significant positive correlation was observed between age and the Reassurance subscale (r = 0.151, *p* < 0.001), indicating that older participants tended to seek more reassurance when searching for health information online. This may reflect greater caution or concern about health issues that can come with increasing age.

### 4.2. The Association of Certain Health-Related Characteristics with Cyberchondria

In this study, individuals with chronic illnesses were found to have higher Cyberchondria Severity Scale (CSS) scores. The literature includes studies that both support this finding [37,44] and report higher scores among those without chronic illnesses [11,16]. Individuals with chronic conditions often use the internet as an affordable, easily accessible, and rapid source of information due to the ongoing nature of their illness and their high information needs; consequently, they tend to engage in online health information-seeking behavior more frequently. On the other hand, individuals who have symptoms but have not yet received a diagnosis may experience higher uncertainty and health anxiety, which can also lead to increased online research tendencies and explain discrepancies in the literature. In this context, enhancing health literacy among individuals with chronic diseases may be a useful strategy associated with reduced cyberchondria behaviors by enabling them to evaluate online health information more consciously and accurately.

In this study, participants who rated their general health status as poor had higher Cyberchondria Severity Scale (CSS) scores compared to those who reported good health, both in bivariate and multivariate analyses (B = −3.065, *p* < 0.001). Similarly, Erdoğan et al. (2020) found that individuals perceiving their health as very poor/poor/moderate had higher CSS scores [40]. Sabir and Naqvi (2023) also reported higher levels of cyberchondria among students who perceived their health status as poor [45]. In the population, individuals with a negative perception of their health may experience a vicious cycle wherein low health perception and increased cyberchondria mutually reinforce each other; heightened health anxiety leads to more frequent online health information seeking, which in turn exacerbates health worries and re-triggers anxiety. Therefore, it is recommended that health literacy programs targeting individuals who negatively evaluate their general health be prioritized. Such interventions can not only improve individuals’ skills in accessing online information but also reduce health-related misconceptions and anxieties, playing a critical role in breaking the cycle of cyberchondria.

In this study, participants who most frequently used the internet to access health-related information were found to have higher Cyberchondria Severity Scale (CSS) scores. Similar findings have been reported in other studies in the literature, supporting this result [37,46]. This finding suggests that increased online health information-seeking behavior may lead to greater anxiety in individuals, thereby increasing the need for frequent information searching. Moreover, factors such as insufficient scientific accuracy, contradictory or incorrect content in online health information may have contributed to this increase. This situation can further exacerbate individuals’ health-related uncertainties, creating a fertile ground for cyberchondria. Therefore, improving the content quality of internet-based health information sources, ensuring the accuracy of the information, and promoting user-friendly and reliable platforms not only facilitate easier access to information but also play a crucial role in preventing the development of cyberchondria.

### 4.3. The Association of Health Literacy with Cyberchondria

In this study, a small negative correlation was found between participants’ health literacy scale scores and their Cyberchondria Severity Scale (CSS) scores. Furthermore, multivariate analysis revealed that an increase in health literacy level was significantly associated with a decrease in cyberchondria severity (B = −0.329, *p* < 0.001). Several studies in the literature examining the relationship between health literacy (HL) and cyberchondria have addressed this association at the bivariate level and similarly reported a negative correlation between HL and cyberchondria [11,26,27,47].

However, cyberchondria is a multidimensional phenomenon influenced by a wide range of factors, including individual health anxieties, demographic characteristics, and health perception. Therefore, analyses focusing solely on the relationship between two variables may be insufficient to explain this complex construct. Kobryn and Duplaga (2024), using multivariate regression analysis, also concluded that higher health literacy levels reduce the risk of cyberchondria [48].

Cyberchondria, although not explicitly recognized in the DSM diagnostic system, is considered a concept related to psychopathological conditions such as hypochondriasis, health anxiety, and generalized anxiety disorder. Within this context, several studies have reported that low health literacy levels are associated with increased health anxiety. For example, a survey conducted during the COVID-19 pandemic among patients attending a clinic in Iran demonstrated a significant association between lower health literacy scores and higher health anxiety levels [49]. Although this study did not directly investigate cyberchondria, considering the overlapping features of related psychological conditions with cyberchondria, these findings provide a foundation for understanding psychiatric and individual factors that may contribute to the development of cyberchondria. Similarly, Ali et al. (2024) showed that health literacy exerts an indirect effect on cyberchondria through health anxiety [50]. Moreover, there is evidence indicating that health literacy reduces cyberchondria indirectly by strengthening the negative relationship between health anxiety and emotional regulation via mediator and moderator effects [51].

These findings indicate that higher health literacy levels are associated with lower severity of cyberchondria, both directly and indirectly. This outcome can be explained by the fact that individuals with higher health literacy possess a greater ability to critically and consciously evaluate online health information. Such individuals are likely to be more resilient against misinformation and able to base their health-related decisions on more reliable grounds. Additionally, they tend to have higher levels of trust in healthcare professionals, which may be associated with less unnecessary online health information-seeking behaviors. It is essential to recognize that health literacy encompasses not only access to information but also cognitive skills such as critical appraisal of information, evaluation of source credibility, and development of appropriate health behaviors. Based on these findings, initiatives aimed at enhancing individuals’ skills to evaluate online health information and expanding programs to strengthen health literacy at the population level may be associated with lower levels of digital behavioral disorders such as cyberchondria.

Numerous studies in the literature examining the impact of e-health literacy on cyberchondria have found that increases in e-health literacy levels are significantly associated with higher levels of cyberchondria [41,48,52,53,54]. In another study investigating the effect of e-health literacy on cyberchondria among healthcare workers, it was reported that increases in the excessiveness subscale scores of the Cyberchondria Severity Scale were statistically linked to higher e-health literacy levels [20]. These findings, which differ from the relationship observed between general health literacy and cyberchondria, suggest that the structural and functional distinctions between health literacy and e-health literacy may be associated with differing patterns of cyberchondria. While health literacy encompasses individuals’ abilities to understand, evaluate, and appropriately use health information, e-health literacy focuses more specifically on individuals’ capacity to access health information in digital environments, filter this information, and effectively utilize digital health resources. Individuals with high e-health literacy may tend to seek online health information more frequently, and when combined with excessive exposure to information, this may increase the risk of cyberchondria. This distinction points to the importance of examining how different types of health literacy relate to cyberchondria. It emphasizes the need for conscious guidance, particularly aimed at the use of digital information.

### 4.4. Limitations

This study has several limitations. The cross-sectional design of the study limits the ability to make precise inferences about the directionality and causality of the relationships between variables. Additionally, since the data were self-reported by participants, responses may be subject to various biases such as social desirability bias and recall bias. In addition, the study was conducted over five consecutive weekdays in a single tertiary hospital outpatient setting using convenience sampling, and participation was restricted to adults who reported using the internet for health-related information. This introduces selection bias, limits the representativeness of the sample, and could limit the generalizability of the findings. Furthermore, the study included only limited measurement of AI-assisted health search, as data were collected in June 2023 when the adoption of AI chat and search tools was emerging. Future studies should consider including items assessing AI/LLM use to better capture its potential influence on cyberchondria. Moreover, the study assessed only general health literacy levels and their association with cyberchondria, without measuring e-health literacy (eHL), which reflects skills related to searching and using health information in digital settings. This represents a potential limitation, especially given the digital behavior-based nature of cyberchondria. Future research is recommended to include more comprehensive evaluations using samples from various institutions and regions, as well as multivariate models incorporating variables such as digital health literacy and health-related anxiety.

## 5. Conclusions

In this study, the relationship between health literacy and cyberchondria was evaluated, revealing a significant negative association: higher health literacy levels were linked to lower levels of cyberchondria. This association remained significant in both univariate and multivariate models that accounted for sociodemographic and health-related factors, indicating that health literacy is independently associated with cyberchondria, although the strength of this association is limited. Additionally, female gender and negatively perceived health status emerged as sociodemographic factors associated with increased cyberchondria.

Based on these findings, community-based educational programs aimed at improving health literacy should be expanded, with priority interventions focusing on women and individuals reporting poor perceived health. Informative content and guidelines focusing on the dimension of digital health literacy should be developed to support individuals’ critical appraisal skills of online health information. Furthermore, it is crucial to establish national health literacy strategies that integrate goals to raise awareness about cyberchondria. Considering the critical role of healthcare professionals in this context, physicians should guide patients in evaluating the health information obtained from the internet and adopt a communication approach that involves jointly verifying the accuracy of such information.

## Figures and Tables

**Table 1:** Some Descriptive Characteristics of Participants and Comparison of Cyberchondria Severity Scale Scores According to These Characteristics.

*n* = 965	*n* (%)	Cyberchondria Severity Scale Score	
		Mean ± SD	*p*
Gender			
Female	531(55.0)	85.9 ± 20.5	0.001
Male	434 (45.0)	81.8 ± 18.1	
Marital Status			
Married	451 (46.7)	85.0 ± 19.4	0.183
Single	514 (53.3)	83.3 ± 19.7	
Educational Status			
Elementary school graduate and below	38 (3.9)	83.3 ± 22.2	0.003 a–b: 0.002
Middle school graduate	54 (5.6)	86.8 ± 20.5	
High school graduate ^a^	318 (33.0)	87.0 ± 19.2	
University graduate or above ^b^	555 (57.5)	82.1 ± 19.3	
Monthly Income Status of the Household			
My expenses exceed my income	229 (23.7)	83.5 ± 19.6	0.276
My income equals my expenses	414 (42.9)	85.2 ± 19.7	
My income exceeds my expenses	322 (33.4)	83.0 ± 19.4	
Chronic Illness Status			
Yes	318 (33.0)	87.2 ± 20.1	0.001
No	647 (67.0)	82.5 ± 19.1	
Presence of Chronic Disease in the Family			
Yes	581 (60.2)	84.2 ± 19.4	0.813
No	384 (39.8)	83.9 ± 20.0	
Perceived Health Status			
Very Bad/Bad ^a^	48 (5.0)	97.4 ± 21.3	<0.001 a–b: <0.001
Middle ^b^	236 (24.5)	84.6 ± 19.4	
Good ^b^	507 (52.5)	84.1 ± 19.1	
Very Good ^b^	174 (18.0)	79.7 ± 19.1	
Most Frequent Use of the Internet When Accessing Health-Related Information			
No	477 (49.4)	80.8 ± 19.0	<0.001
Yes	488 (50.6)	87.2 ± 19.6	

^a–b^: Post Hoc test results.

**Table 2:** The Correlation Between Participants’ Age and Health Literacy Scale Scores and Cyberchondria Severity Scale Scores.

*n* = 965		Age	Health Literacy (HL) Scale Score
Age	r	1	
	*p*		
Health Literacy (HL) Scale Score	r	−0.229 *	1
	*p*	<0.001	
Cyberchondria Severity Scale (CSS) Score	r	0.033	−0.165 *
	*p*	0.312	<0.001
CSS Compulsion Subscale	r	−0.003	−0.244 *
	*p*	0.920	<0.001
CSS Distress Subscale	r	0.063	−0.186 *
	*p*	0.049	<0.001
CSS Excessiveness Subscale	r	−0.054	0.012
	*p*	0.092	0.704
CSS Reassurance Subscale	r	0.151 *	−0.041
	*p*	<0.001	0.203
CSS Mistrust Of Medical Professional Subscale	r	−0.042	−0.291 *
	*p*	0.192	<0.001

*: The correlation significance level is 0.01.

**Table 3:** Multiple Linear Regression Analysis of Factors Associated with the Level of Cyberchondria.

MODEL		Unstandardized Coefficients B (Std. Error)	Standardized Coefficients Beta	*p*
1	CONSTANT	96.264 (2.433)		<0.001
	Health Literacy Score (HLS)	−0.376 (0.072)	−0.165	<0.001
2	CONSTANT	94.039 (2.510)		<0.001
	Health Literacy Score (HLS)	−0.378 (0.072)	−0.166	<0.001
	Gender (ref. male)	4.167 (1.244)	0.106	0.001
3	CONSTANT	101.180 (3.124)		<0.001
	Health Literacy Score (HLS)	−0.329 (0.073)	−0.144	<0.001
	Gender (ref. male)	4.095 (1.236)	0.104	0.001
	Perceived Health Status (ref. very bad/bad)	−3.065 (0.808)	−0.121	<0.001

Notes: Perceived health status was entered as an ordinal variable (1 = very bad, 4 = very good); the coefficient represents the expected change in cyberchondria score for each one-level increase in perceived health status.

## Data Availability

All the data in the article are available from the corresponding author upon reasonable request.

## Abbreviations

The following abbreviations are used in this manuscript:

eHL	e-health literacy
HLS-SF	Short Form Health Literacy Scale
CSS	Cyberchondria Severity Scale
